# Evolutionary patterns of *Escherichia coli* small RNAs and their regulatory interactions

**DOI:** 10.1261/rna.043133.113

**Published:** 2014-07

**Authors:** Asaf Peer, Hanah Margalit

**Affiliations:** Department of Microbiology and Molecular Genetics, Faculty of Medicine, The Hebrew University of Jerusalem, Jerusalem 91120, Israel

**Keywords:** small RNA, evolutionary reconstruction, phylogenetic tree, post-transcriptional regulation, bioinformatics

## Abstract

Most bacterial small RNAs (sRNAs) are post-transcriptional regulators of gene expression, exerting their regulatory function by base-pairing with their target mRNAs. While it has become evident that sRNAs play central regulatory roles in the cell, little is known about their evolution and the evolution of their regulatory interactions. Here we used the prokaryotic phylogenetic tree to reconstruct the evolutionary history of *Escherichia coli* sRNAs and their binding sites on target mRNAs. We discovered that sRNAs currently present in *E. coli* mainly accumulated inside the *Enterobacteriales* order, succeeding the appearance of other types of noncoding RNAs and concurrently with the evolution of a variant of the Hfq protein exhibiting a longer C-terminal region. Our analysis of the evolutionary ages of sRNA–mRNA interactions revealed that while all sRNAs were evolutionarily older than most of their known binding sites on mRNA targets, for quite a few sRNAs there was at least one binding site that coappeared with or preceded them. It is conceivable that the establishment of these first interactions forced selective pressure on the sRNAs, after which additional targets were acquired by fitting a binding site to the active region of the sRNA. This conjecture is supported by the appearance of many binding sites on target mRNAs only after the sRNA gain, despite the prior presence of the target gene in ancestral genomes. Our results suggest a selective mechanism that maintained the sRNAs across the phylogenetic tree, and shed light on the evolution of *E. coli* post-transcriptional regulatory network.

## Abstract (precis)

This article describes an evolutionary analysis of small regulatory RNAs (sRNAs) in *E. coli*. Findings include the observation that sRNAs are older than their known binding sites on target mRNAs. Many current targets were acquired by evolving binding sites that pair with the active regions of sRNAs.

## INTRODUCTION

It is now well established that many transcribed genes are not further translated to proteins but rather function in the cell as noncoding RNAs (ncRNAs). Classical ncRNAs, for example, are the widely known tRNAs and rRNAs that play a role in translation, or the tmRNA that plays a role in the rescue of stalled ribosomes (Janssen and Hayes 2012). During recent years it has become evident that the ncRNA world is substantially wider and includes other types of molecules. In particular, small regulatory RNAs involved in the post-transcriptional regulation of gene expression were discovered in all kingdoms of life and were shown to play important roles in the regulation of central cellular processes (for reviews, see Bartel 2009; Carthew and Sontheimer 2009; Waters and Storz 2009).

In bacteria, ncRNAs are utilized in various ways to regulate mRNA and protein levels of many genes. In general, regulatory ncRNAs can be classified into two types. The first type involves RNA elements that are transcribed as part of their target gene (usually at the 5′ end) to regulate its transcription or translation, referred to as *cis* regulators. They include, for example, attenuators, which change the mRNA secondary structure to allow or terminate transcription, depending on the metabolic state of the cell (Naville and Gautheret 2009), and riboswitches, which regulate translation initiation in response to altered temperature or by sensing a specific ligand (Breaker 2011). The second type of regulatory ncRNAs, referred to as *trans*-acting ncRNAs, includes RNA elements that regulate the expression of genes transcribed elsewhere on the chromosome. This class includes the small RNAs (sRNAs), which are 50- to 400-nucleotide (nt)-long molecules encoded in the genomes of many bacteria (Storz et al. 2011). They do not make part of an adjacent mRNA but are transcribed into independent transcripts that most often have a post-transcriptional regulatory function. Most of the studied sRNAs are negative regulators that were shown to down-regulate gene expression by base-pairing with their target mRNAs, either interfering with ribosome binding or destabilizing the mRNA (Storz et al. 2011). Several sRNAs were shown to positively regulate gene expression by base-pairing with the mRNA, enhancing translation by exposing a hitherto occluded ribosome binding site and/or by stabilizing the mRNA (Majdalani et al. 1998; Papenfort et al. 2013). In many bacteria, including *Escherichia coli*, the interaction between the sRNA and the mRNA of its target gene is mediated by the Sm-like protein Hfq, which binds both RNAs (for reviews, see Brennan and Link 2007; Vogel and Luisi 2011).

In the current study, we focus on the *trans*-acting sRNAs that exert their regulatory function by base-pairing with their target mRNAs. While it has become evident that these molecules play central regulatory roles in the cell, little is known about their evolution and about the evolution of the regulatory interactions they are involved in. Skippington and Ragan (2012) analyzed the conservation of sRNAs and their target genes within the clade of *Shigella* and *E. coli*, and defined core and variable sRNAs and targets. Here we took a broader view and reconstructed the evolution of *E. coli* sRNAs and their regulatory interactions throughout the bacterial phylogenetic tree, taking advantage of the recently deciphered genome sequences of numerous bacteria. Our analysis has revealed that the sRNAs of *E. coli* accumulated mostly inside the *Enterobacteriales* order, after the appearance of *cis*-acting and housekeeping ncRNAs and concurrently with the evolution of a variant of the Hfq protein exhibiting a longer C-terminal region. By tracing the evolution of *E. coli* sRNAs along with their respective binding sites on target mRNAs, we determined different scenarios for their order of appearance. Our results support an evolutionary model by which the establishment of a regulatory interaction between a sRNA and a mRNA forced selective pressure on the sRNA, followed by accumulation of additional targets that evolved binding sites fitting the sRNA (Gottesman and Storz 2011; Richter and Backofen 2012).

## RESULTS

### Evolution of *E. coli* sRNAs

We traced the evolution of ncRNA families found currently in *E. coli*. A ncRNA family contains ncRNA genes that share a consensus secondary structure and show sequence similarity, as defined in the Rfam database (Burge et al. 2013). For example, all the 86 tRNA genes of *E. coli* are included within one Rfam family, while the families of most sRNAs are of size 1. In total, our data set contained 102 *E. coli* Rfam families, which we manually clustered into three groups (Supplemental Table S1): (1) the group of *cis*-acting ncRNAs, which contained 31 ncRNA families, including riboswitches, attenuators, and leader sequences, regulating the transcription or translation of their adjacent genes; (2) the group of *trans*-acting ncRNAs, which included 58 ncRNA families (sRNAs), regulating their target genes post-transcriptionally by base-pairing with their mRNAs; and (3) the group of other ncRNAs, which included all the rest of the ncRNA families (13 families), among them were the rRNAs and tRNAs, as well as the protein-binding sRNA families (CsrB/CsrC).

**FIGURE 1.**
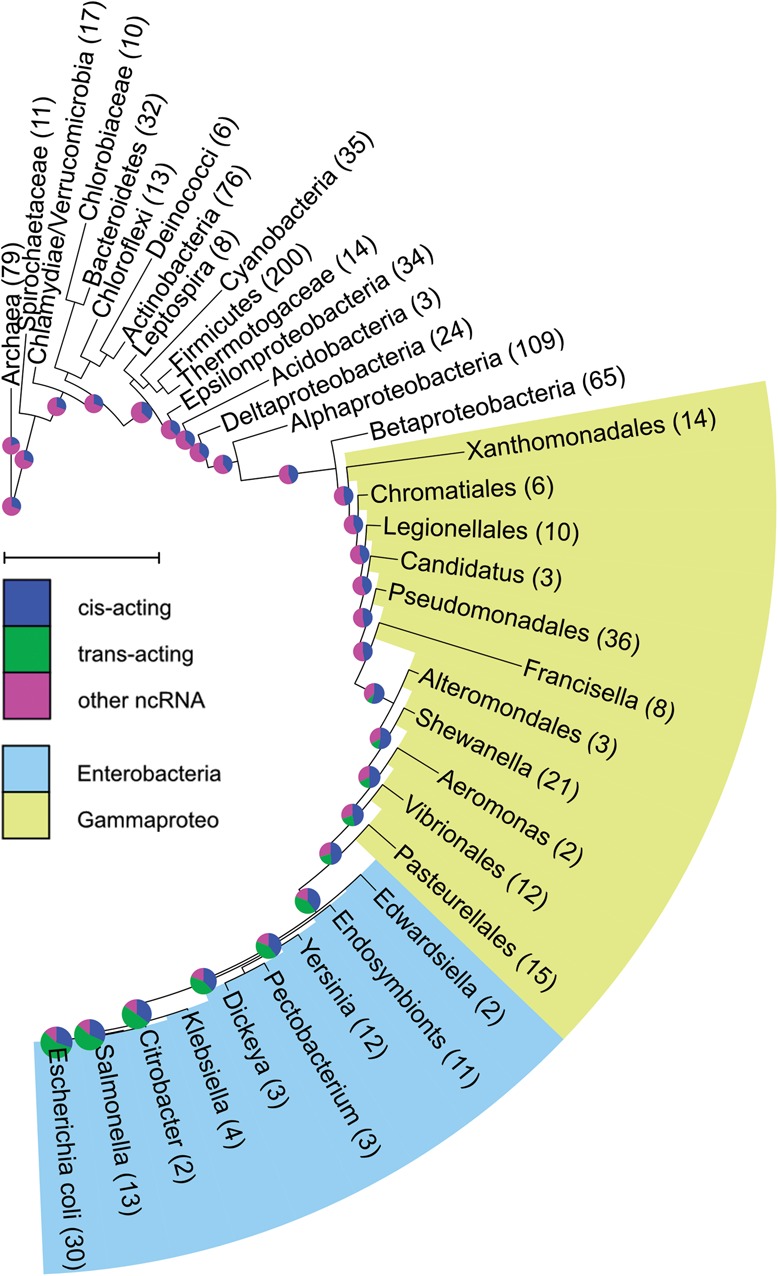
Distribution of *E. coli* ncRNA families in ancestral genomes. Evolution of ncRNA families, as defined by Rfam, was reconstructed on the phylogenetic tree of prokaryotes. For each ancestral genome of *E. coli*, the distribution of the three groups of ncRNA families is represented as a pie chart (green indicates *trans-*acting ncRNAs [sRNAs]; blue, *cis-*acting ncRNAs; magenta, other ncRNAs). The size of each pie chart is proportional to the number of its total Rfam ncRNA families, ranging from 13 in the oldest ancestor to 102 in *E. coli*. It can be easily seen that most *trans*-acting sRNAs of *E. coli* first appeared inside the γ-proteobacteria class (colored in light green), where most of them appeared when the *Enterobacteriales* order split from the rest of the γ-proteobacteria and inside the *Enterobacteriales* (colored in light blue). The numbers in parentheses represent the number of genomes in each clade used for the reconstruction (e.g., the *E. coli* clade, including also *Shigella*, contained 30 genomes); scale bar, an evolutionary distance of 0.5-amino-acid substitutions per position. The figure was prepared using iTOL (Letunic and Bork 2007, 2011).

For each ncRNA family in the three groups, we first determined the prokaryotic genomes that encode at least one representative of the family, using the information in the Rfam database. Accordingly, we annotated each leaf node of the prokaryotic phylogenetic tree as possessing or lacking this ncRNA family and carried out evolutionary reconstruction using a maximum likelihood approach implemented in GLOOME (Cohen and Pupko 2010). Based on this reconstruction, we determined for each ncRNA family the most likely evolutionary scenario and the ancestral node where it was gained. The distance from a respective gain node to the *E. coli* node on the tree determined the evolutionary age of the ncRNA family (Supplemental Tables S1, S2). By examining the ncRNA family repertoire in the ancestral genomes of *E. coli*, we could follow the order of appearance of the various types of ncRNA families. Our analysis revealed that the “other ncRNAs” appeared first, along with several *cis*-acting ncRNA families that continued to accumulate later on, while the *E. coli trans*-acting sRNAs were the latest to appear in evolution (*P*< 7 × 10^−5^ in two-tailed Mann-Whitney test comparing the evolutionary ages of *cis*- and *trans*-acting ncRNAs) (Fig. 1; Supplemental Table S1). Furthermore, as is clearly observed in Figure 1, there has been substantial accumulation of the *trans-*acting sRNA genes found currently in *E. coli* when the *Enterobacteriales* order split from the rest of the γ-proteobacteria and inside the *Enterobacteriales* order, whereas the *cis*-acting ncRNAs accumulated evenly along the branches of the tree. Similarly, a remarkable fraction of *Vibrio* sRNAs were shown to be specific to the *Vibrio* genus (Toffano-Nioche et al. 2012), suggesting that the lineage-specific accumulation of sRNAs is not specific to the enterobacteria. Of note, there were eight exceptions of *E. coli* sRNAs that were present outside of the *Enterobacteriales* order, including four sRNAs with known targets. These sRNAs are Spot-42, GcvB, RyhB, and SgrS, which are involved in the regulation of sugar metabolism, amino-acid import, iron storage and metabolism, and sugar uptake, respectively.

Because Rfam defines a family of ncRNAs using a combination of structure and sequence information formulated as a covariance model (Eddy and Durbin 1994), it is possible that the definition of some *cis-*regulatory RNA families is more amenable since their structure is important for their function, and thus they can be more easily identified, resulting with representatives in more genomes. In order to validate that the previous result is not biased, we repeated the analysis, this time using annotations of the *E. coli* ncRNA families in other genomes based on BLAST searches (Altschul et al. 1990). For each ncRNA gene, we took the sequences of all the family members in *E. coli*, searched for them in the prokaryotic genomes represented in the phylogenetic tree using BLAST, and annotated the genomes accordingly (Materials and Methods). We reconstructed the evolution of each ncRNA family based on this new annotation and determined their evolutionary ages. The ages obtained using BLAST-based annotations were in good agreement with the ones obtained using Rfam-based annotations for the *trans*-acting sRNAs (Supplemental Table S1), while *cis*-acting ncRNAs appeared to be younger by the reconstruction following the BLAST-based annotations compared with the Rfam-based annotations because BLAST failed to identify some of them in remote bacteria. Still, this latter analysis, in which the ncRNA genes along the tree were identified by sequence considerations only, reinforced our finding that the *trans*-acting ncRNAs are younger than the *cis*-acting ncRNAs (*P* = 0.0027 using two-tailed Mann-Whitney test) (Supplemental Table S1).

The accumulation of sRNAs found currently in *E. coli* in the *Enterobacteriales* order probably gave bacteria in this order some advantages. While it is not clear what has enabled this substantial sRNA accumulation, we suggest that it might be associated with the evolution of the Hfq protein. It was shown that the Hfq variant of enterobacteria contains a C-terminal region (CTR) that is longer than that of other γ-proteobacteria, such as Vibrio (Vincent et al. 2012). We examined the amino acid sequences of Hfq in γ-proteobacteria and found that indeed all enterobacteria have a relatively long CTR with >60% similarity to that of *E. coli*. Our reconstruction of this long CTR has revealed that it was gained in the *Enterobacteriales* split (Supplemental Fig. S1), suggesting that it might play a role in the concurrent accumulation of the sRNAs. Yet, there are contradicting findings regarding the role of the extended CTR of Hfq in sRNA–mRNA interaction. While some studies showed that this longer CTR makes the Hfq hexamer more stable, enables it to bind double-stranded RNA, and is required for regulation by sRNAs (Vecerek et al. 2008; Beich-Frandsen et al. 2011; Vincent et al. 2012), other studies demonstrated that deleting this long C-terminal tail has no effect on sRNA binding and riboregulation (e.g., Olsen et al. 2010). Notwithstanding this controversy regarding the functional importance of the long CTR, its conservation in the *Enterobacteriales* order may suggest that it has been conserved for a functional reason, which might be associated with the accumulation of the sRNAs found currently in *E. coli* inside this order.

### Evolution of sRNA–mRNA regulatory interactions

A regulatory interaction between a sRNA and a mRNA involves base-pairing between the active region of the sRNA and its binding site on the mRNA. Thus, a regulatory interaction presently observed in *E. coli* could have been established in ancestral genomes across the evolutionary tree when both binding sites, the binding site on the sRNA (sBS) and the binding site on the target mRNA (mBS), have evolved. We can study the evolutionary scenarios that underlay the establishment of these interactions by following the evolution of the sRNA and sBS and the target gene and mBS. To this end we used a data set of 60 sRNA–mRNA interactions in *E. coli*, which were experimentally determined. Some interactions were determined in *Salmonella* and were included here because the interacting nucleotides were found to be fully conserved in *E. coli*, suggesting that the regulation is valid there too. In total, the regulatory interactions involved 15 sRNAs and 49 target genes (Supplemental Table S2 and references therein).

We first turned to follow the evolution of sRNAs and sBSs. We already reconstructed the evolutionary history of the *E. coli* sRNAs using the prokaryotic phylogenetic tree (see above). However, while in the above analysis we considered the maximum likelihood reconstruction, in the analysis of the sRNAs in respect to their target genes and mBSs, we wished to take a more stringent approach, ascertaining the presence of the studied elements in the reconstructed gain node and avoiding inconclusive results. The advantage of using GLOOME in this respect is the posterior probabilities it provides for each internal node to have a studied trait. GLOOME uses a stochastic mapping approach to model gain and loss events along the phylogenetic tree. The parameters for the model are estimated using maximum likelihood, and then the gain and loss posterior probabilities for each trait along each branch of the tree can be estimated. These can be used to evaluate the posterior probability of each internal node in the tree to possess the trait. Thus, in the current analysis we determined a gain node when its posterior probability to have the sRNA (or any other element in the following analysis) was 0.7 or higher. Of note, the two approaches for determining the gain nodes (in the current and previous analysis) yielded consistent evolutionary ages, except for SgrS (which was determined as ancient sRNA above and here was found to appear in the *Enterobacteriales* order). In the latter reconstruction, SgrS was found to be gained in two nodes, hinting at a potential horizontal gene transfer that took place, which might cause the inconsistency in reconstruction by the two approaches. Hereinafter we consider SgrS by the age determined by the latter analysis in the branch of *E. coli*, leaving three ancient sRNAs among the 15 analyzed here: Spot-42, RyhB, and GcvB.

To determine the ages of the sBSs, we should have carried out a similar reconstruction, using the sBS sequences. However, in previous studies we and others found that the sBSs are highly conserved compared with other sequence regions of *E. coli* sRNAs (Peer and Margalit 2011; Richter and Backofen 2012), implying that they are present along with the presence of the sRNA across the phylogenetic tree. Here we further verified by sequence alignments and evolutionary reconstruction carried out by the prank algorithm (Löytynoja and Goldman 2005) that the reconstructed sBS sequence in the gain node of the sRNA is highly similar to the sBS sequence of *E. coli.* In fact, we found for almost all sBSs that the corresponding sequences in the gain node and in *E. coli* are identical. Therefore, we regarded the gain node of the sRNA as the gain node of its sBS(s) (Supplemental Table S2).

We next turned to trace the evolution of each target and its mBS(s). To this end, we first fetched the target gene orthologs in genomes along the tree from the oma-browser database (http://omabrowser.org/; Dec-2012 version) (Altenhoff et al. 2011). We then identified for each target ortholog the region corresponding to the *E. coli* mBS by sequence alignment. Having determined in each genome the corresponding sBS and putative mBS, we further substantiated the mBS as involved in a regulatory interaction in the studied genome by its potential base-pairing with the sBS, as evaluated by free energy computation (Materials and Methods). Finally, we reconstructed the evolution of the target gene and of the mBS using the GLOOME algorithm (Cohen and Pupko 2010). This has allowed us to identify the ancestral genomes harboring the target gene and to specify those that contained the mBS. Based on these assignments, we determined the gain nodes of the target genes and respective mBSs and, hence, their evolutionary ages.

**FIGURE 2.**
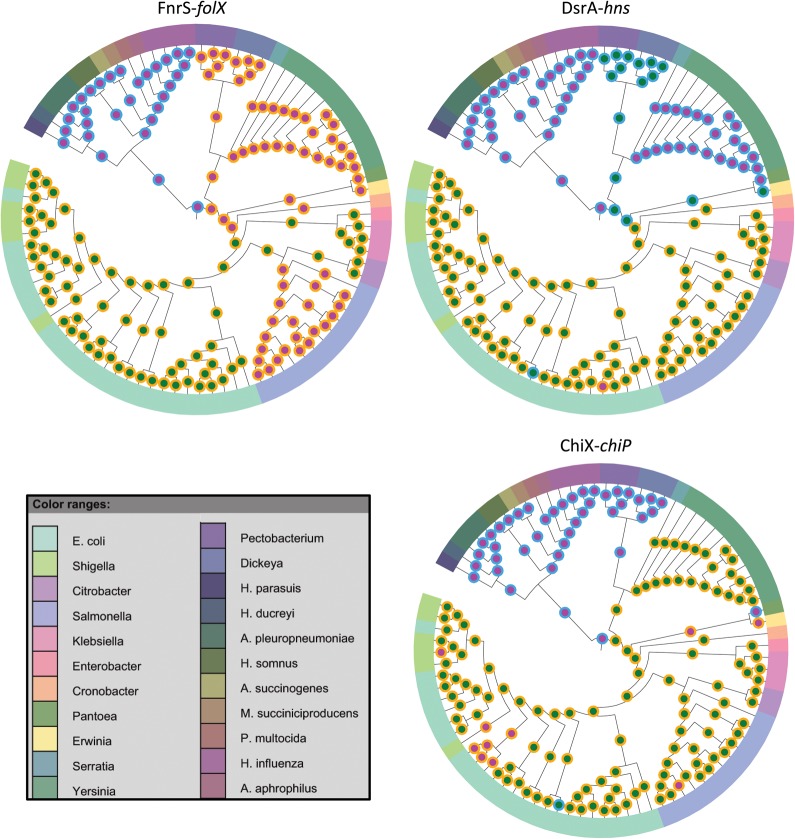
Order of appearance of sRNAs and their binding sites. Three examples of the evolutionary scenarios of the appearance of a sRNA and one of its mBSs are shown. Each tree represents a subset of the γ-proteobacteria class. The outer circle at each node represents the sRNA: It is blue when the sRNA is absent from the genome and orange when it is present. The inner circle represents the mBS: It is magenta for absence and green for presence of the mBS. In the FnrS-*folX* example, the sRNA appeared before the mBS; in DsrA-*hns*, the sRNA was gained after the mBS; and in the ChiX-*chiP* example, the binding site and the sRNA were co-gained along the same branch of the tree. The figures were generated using iTOL (Letunic and Bork 2007, 2011). Branch lengths are not to scale.

**FIGURE 3.**
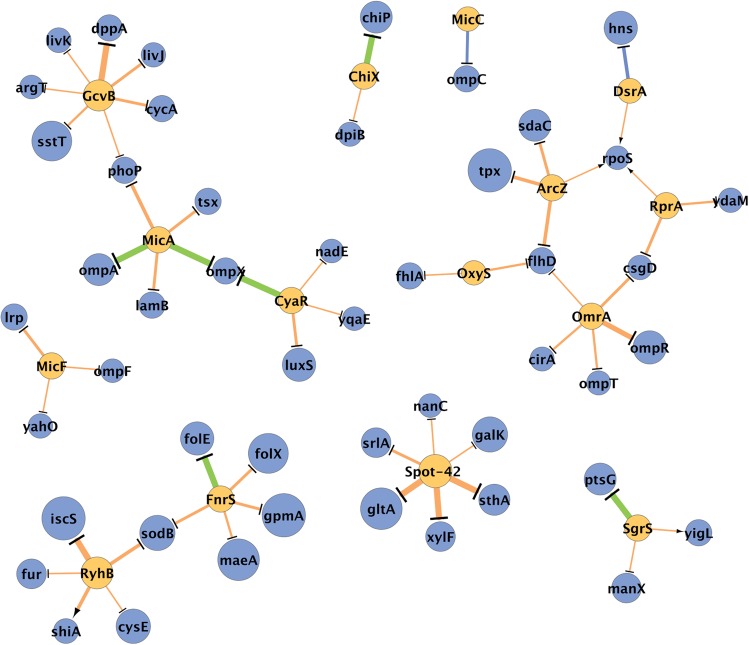
Evolution of the post-transcriptional regulatory network. The presented network includes the experimentally determined interactions studied here. Nodes are sRNAs and targets, and edges represent interactions. Node size is proportional to its evolutionary age, and the thickness of an edge is proportional to the age of the established interaction (determined by the age of the younger node between the sBS and mBS). The larger the node, the older it is, and the thicker the edge, the older it is. The color of an edge represents the age relationship between the sBS and mBS: blue indicates the mBS preceded the regulating sRNA; green, the mBS coappeared with the regulating sRNAs; and orange, the mBS succeeded the regulating sRNA. The figure was prepared using Cytoscape (Shannon et al. 2003).

**FIGURE 4.**
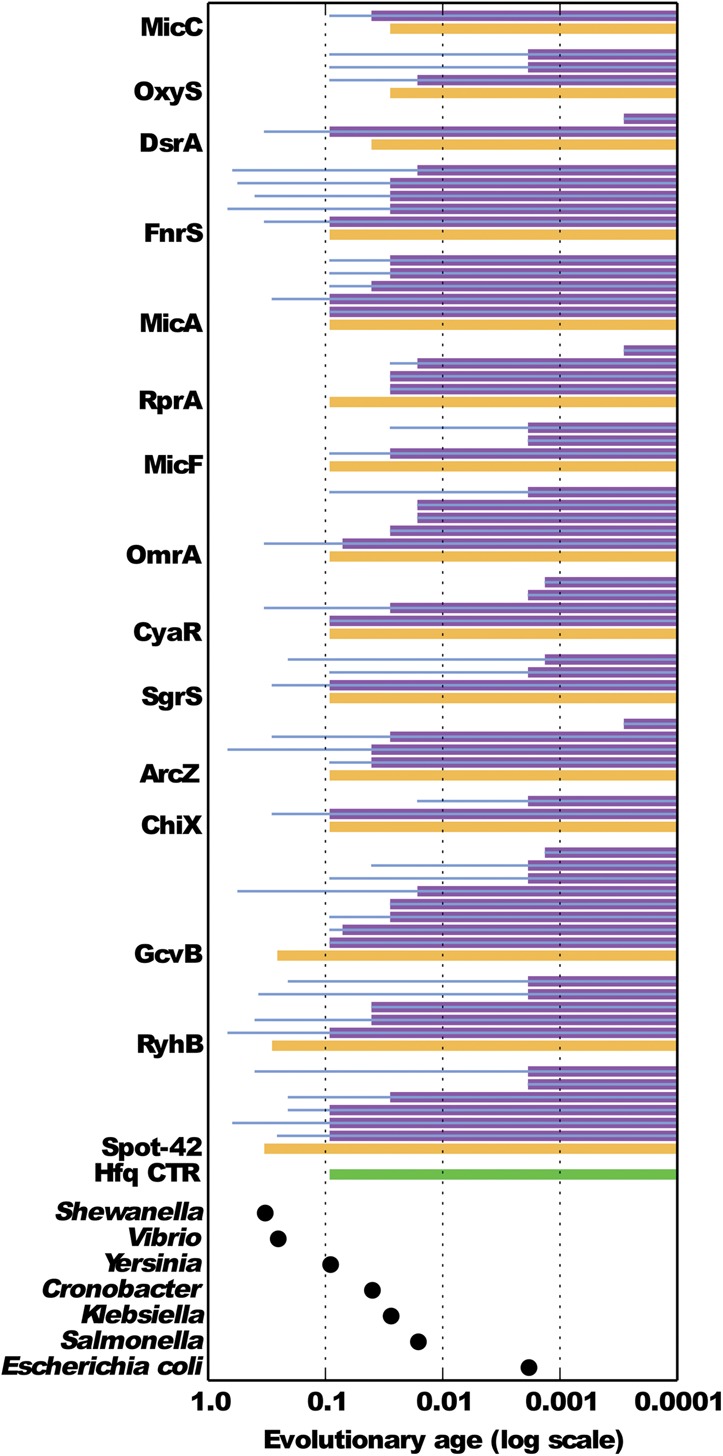
Overview of the evolutionary ages of the sRNAs, their target genes, and mBSs. Presented are the ages of sRNAs (yellow bars), mBSs (purple bars *above* each sRNA bar), and target genes bearing the mBSs (blue lines *within* the purple bars). The evolutionary age of the conserved long CTR of Hfq is presented also (green). The longer the bar, the older the corresponding genetic element is. The ages of the most recent common ancestors of *E. coli* K12 substrain MG1655 and the specified bacteria are marked at the *bottom* of the figure (black dots). For instance, the common ancestor of *E. coli* and *Yersinia*, which is the ancestor of all enterobacteria, has the age of 0.09, while the ancestor of all *E. coli* strains has the age of 0.00184. The *x*-axis is in log scale.

The age of a regulatory interaction is determined by the younger node between the gain nodes of the corresponding sBS and mBS. There are three possible scenarios for interaction establishment: (1) the sBS and mBS were co-gained in the same ancestral node; (2) the mBS preceded the sBS; and (3) the mBS succeeded the sBS. In Figure 2 we demonstrate detailed examples of these various evolutionary scenarios. Examination of these scenarios in our data set revealed that the mBS succeeded the sBS in 52 of the 60 interactions (Figs. 3, 4; Table 1; Supplemental Table S2). In six of the remaining interactions, we found that the mBS and sBS were co-gained (ChiX-*chiP*, CyaR-*ompX*, FnrS-*folE*, MicA-*ompA*, MicA-*ompX*, and SgrS-*ptsG*), and in two other interactions, the mBSs preceded the sBSs (DsrA-*hns* and MicC-*ompC*). These eight latter interactions involved seven of the 15 studied sRNAs. Thus, for ∼50% of the studied sRNAs, the gain of the sRNA was closely accompanied by the establishment of a regulatory interaction, providing the sRNA a function that enhanced its maintenance across the phylogenetic tree. Three of the sRNAs for which all mBSs succeeded the sBS are the relatively ancient sRNAs, Spot-42, GcvB, and RyhB. Interestingly, all their mBSs (19 in total) were established after the appearance of the longer version of the Hfq protein in the *Enterobacteriales* order (Fig. 4; Supplemental Table S2).

**TABLE 1.**
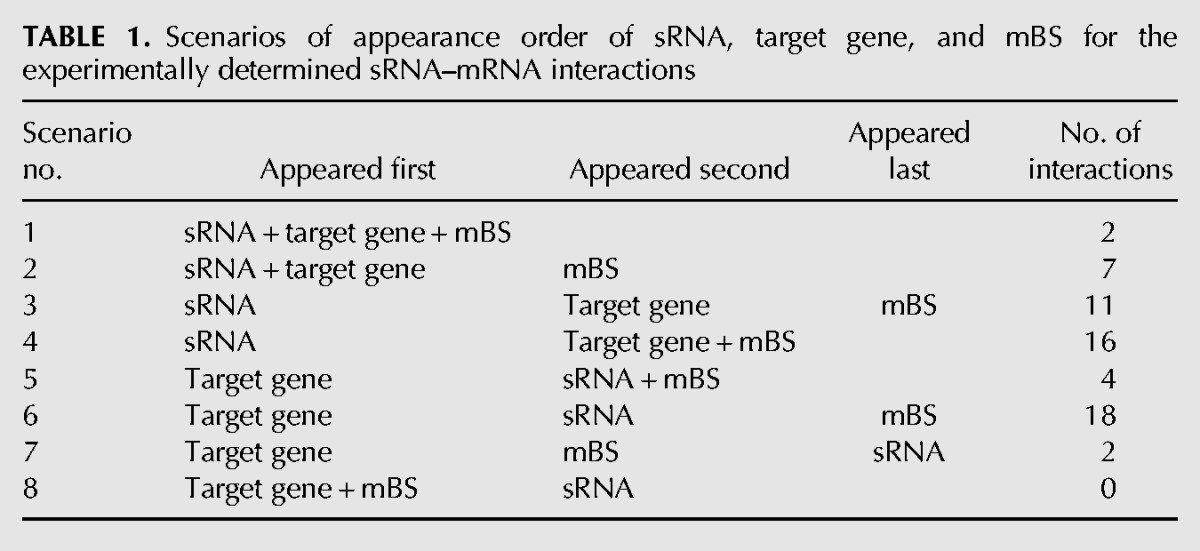
Scenarios of appearance order of sRNA, target gene, and mBS for the experimentally determined sRNA–mRNA interactions

In the analysis above, the ages of mBSs and sRNAs (sBSs) were compared. It is also informative to add to this analysis the evolutionary history of the target genes containing the mBSs (Table 1; Supplemental Table S2). Obviously, the mBS has appeared either along with or subsequent to the appearance of the target gene. Our analysis revealed that for 18 interactions, the mBS appeared along with the gene, and for 42 interactions, the mBS succeeded the appearance of the gene. Interestingly, in 22 of these 42 interactions, the target gene appeared before the sRNA, but the respective mBS either appeared along with or succeeded the regulating sRNA (four and 18 mBSs, respectively). The appearance of many mBSs only after the sRNA gain, despite the prior presence of the target gene in older genomes, suggests that they evolved in accord with the sRNAs.

## DISCUSSION

It is likely that for a sRNA to be maintained in evolution, it has to have a function it is selected for. Since the function of most sRNAs relies on their base-pairing with their targets, it was suggested that establishment of a regulatory interaction between a sRNA and a mRNA should impose a selective pressure to maintain the sRNA (Gottesman and Storz 2011; Richter and Backofen 2012). Indeed, our systematic analysis supports this model for ∼50% of the studied sRNAs (seven of 15), for which the following two evolutionary scenarios hold. By the first scenario, the sRNA and the binding site on the mRNA coevolved at the same evolutionary time, enabling the regulatory interaction. By the second scenario, the binding site on the mRNA might have been under selective pressure for some other purpose it served, and when a sRNA with a complementary sequence was gained, the interaction was established. All these seven sRNAs (ChiX, CyaR, FnrS, MicA, SgrS, DsrA, and MicC), as well as most of the other sRNAs, were gained in the *Enterobacteriales* order concurrently with the evolution of a possibly more stable variant of the Hfq protein, which might have supported the establishment of the interactions. The five other sRNAs (ArcZ, MicF, OmrA, OxyS, and RprA), which also appeared in the *Enterobacteriales* order, had only younger binding sites on their targets (a total of 19 binding sites). While it is possible that other selective forces maintained these latter sRNAs until the establishment of their first regulatory interaction, it is as well plausible that they did have older binding sites but that these binding sites were either lost from the genome of *E. coli* or were not yet discovered, and therefore we could not trace them. Thus, establishment of an interaction between a gained sRNA and a mBS that either was already present or was gained alongside the sRNA put the gained sRNA under selective pressure to be maintained. This further allowed the acquisition of other targets, which are mRNAs that evolved binding sites matching the sRNA active regions. Indeed, as seen in Figure 4, for all sRNAs there has been gradual accumulation of additional binding sites since the establishment of the interaction with the first mBS. This evolutionary model is further reinforced by our finding that about one-third of the target genes evolved before their regulating sRNA, but their respective mBSs appeared only after their regulating sRNAs were gained.

The three relatively ancient sRNAs in our data set, Spot-42, GcvB, and RyhB, provide interesting insights. All the 19 interactions involving these sRNAs were established after the *Enterobacteriales* order split from the rest of the γ-proteobacteria, when the longer variant of Hfq evolved. Furthermore, in several cases, such as GcvB-*sstT*, both the sRNA and target gene were present along long evolutionary times, but the mBS has not evolved until the *Enterobacteriales* order. While there has been some debate as to the functionality of the longer C-terminal region of Hfq in enterobacteria (Vogel and Luisi 2011), the accumulation of the mBSs of these ancient sRNAs in the *Enterobacteriales* order might support the importance of this region for sRNA–mRNA interactions (Vecerek et al. 2008; Beich-Frandsen et al. 2011). Also, the longer version of Hfq might bind target mRNAs with different specificities than the shorter version (Beich-Frandsen et al. 2011). Indeed, it has been shown, for instance, that RyhB of *Vibrio cholera* regulates the expression of several targets, including *sodB*, which is regulated by RyhB in *E. coli* as well. However, the interaction between RyhB and *sodB* mRNA is mediated through other regions of the sRNAs in the two organisms (Davis et al. 2005), suggesting that the different variants of Hfq might have different binding preferences, which might have supported the regulatory interactions for these sRNAs and, consequently, their maintenance inside the *Enterobacteriales* order.

In a previous work, Richter and Backofen (2012) showed that, on average, the mBSs are not evolutionarily conserved, unlike the sBSs (Peer and Margalit 2011; Richter and Backofen 2012). They attempted to explain the lack of conservation of mBSs, as opposed to that of sBSs, by suggesting that targets and mBSs can be gained and lost, but since the sRNA regulates several genes through the sBS, its sequence should not change. Consistently, we found that many mBSs are relatively young, highlighting the dynamic nature of the post-transcriptional regulatory network. It is also plausible that there was also turnover of binding sites, as there are cases where the binding sites are present on the orthologous mRNA but in positions different than those corresponding to the binding sites in *E. coli* (Wright et al. 2013). While such cases are not regarded in our study, as we are interested in the evolution of the actual sites found presently in *E. coli*, taking them into account may improve target predictions. Indeed, CopraRNA, a recently introduced target prediction algorithm, searches for conserved interactions rather than for conserved binding sites, allowing the interactions to be mediated by different sites (Wright et al. 2013). This algorithm was shown to outperform traditional target prediction algorithms like IntaRNA (Busch et al. 2008) or TargetRNA (Tjaden 2008).

There are several caveats in our analysis and the conclusions drawn. First, the downside of using solely sRNAs from *E. coli* is the inability to determine loss events of the sRNAs along the tree. It is probable that sRNAs were gained and lost along evolution and that targets and binding sites for these sRNAs were also gained and lost. If more data regarding sRNAs and their targets in species outside of the *Enterobacteriales* order were available, we could have spotted loss events that happened ancestrally to *E. coli*. Since such data are scarce, we cannot determine if the accumulation of sRNAs in the *Enterobacteriales* order, for instance, is due to increased gain rate, reduced loss rate, or merely a reflection of the turnover rate. The same is applicable to the accumulation of mBSs, which might have been gained and lost in ancestral genomes. Second, our analysis is based on the current knowledge of experimentally determined mBS–sBS interactions in *E. coli* and several interactions from *Salmonella* that are fully conserved in *E. coli*. Obviously, this network of interactions is far from being complete, and our conclusions might be biased. The network involves only 15 sRNAs out of more than 100 sRNAs discovered in *E. coli* (Raghavan et al. 2011), and it is possible that other *E. coli* sRNAs might exhibit a different evolutionary model. However, since we have not selected these 15 sRNAs in any manner and since they cover a wide range of functionalities and expression conditions, we believe they make up a representative sample of *E. coli* sRNAs. Also, our study involves only 60 already deciphered interactions of these 15 sRNAs, while they are probably involved in many more interactions yet to be discovered. Still, the additional interactions that might be discovered can further strengthen and support the suggested model, as they can add interactions that coappeared with or preceded the sRNAs. Third, as our conclusions rely on the computational reconstruction of the evolutionary history of the sRNAs, target genes, and binding sites, it is possible that a different reconstruction might lead to other insights. The advantage of the GLOOME algorithm used by us is that it provides a posterior probability for each ancestral node to encode a trait. In our analysis we declared a node as a gain node if this probability was 0.7 or greater when analyzing the mRNA–sRNA interactions, while disregarding this probability and relying only on the maximum likelihood reconstruction when reconstructing the evolution of the different ncRNA families. Importantly, using these different reconstruction methods, we observed minor changes in the resulting evolutionary ages, such as observed for SgrS. It is of note that we repeated the analysis of sBS–mBS interactions with higher posterior probability thresholds of 0.8 and even 0.9 and found that it affected the ages of very few sRNAs and mBSs in our data set, supporting the same evolutionary model.

The differences we observed between the evolutionary ages of target genes and the mBSs embedded in them, especially in view of the age of the respective sRNA, highlight the importance and relevance of analysis at the binding site resolution. Thus, we provide a rich resource that enables taking a detailed evolutionary view of the post-transcriptional regulatory network (Fig. 3). It is possible to take a target-centered view and follow the evolution of different binding sites of various sRNAs regulating the same target gene or of the same sRNA binding the mRNA simultaneously in two sites. For example, *sodB* is targeted by both RyhB and FnrS. These interactions involve different regions of *sodB* mRNA, and the interaction with RyhB was first to occur. Likewise, it is possible to take a sRNA-centered view and follow the order of appearance of all known interactions of a sRNA. For example, we found that the earliest interactions of Spot-42 involved *gltA*, *sthA*, and *xylF* mRNAs, and its latest interactions were with *galK* and *nanC* mRNAs. It is also possible to focus on a subset of sRNAs of a certain function or on a defined subset of interactions. This resource (http://margalit.huji.ac.il/evo-sRNA/) can be updated and reanalyzed as the data of deciphered sRNA–target interactions expand, either verifying or revising the suggested evolutionary model. Finally, the framework we present, of the analysis of the binding sites in addition to regulator–target relations, can be applied to other cellular networks as well.

## MATERIALS AND METHODS

### Reconstructing the evolution of ncRNA families

The list of ncRNA families was taken from Rfam (http://rfam.sanger.ac.uk/), version 11.0 (Burge et al. 2013). Each family found in *E. coli* K12 substrain MG1655 was classified by us into one of four groups: *cis-*acting ncRNAs, *trans-*acting ncRNAs (sRNAs), antisense ncRNAs, and rest of ncRNAs. The antisense ncRNAs were excluded from the analysis. We used Rfam annotations to mark the leaf nodes of the prokaryotic phylogenetic tree as possessing or lacking a ncRNA family. The phylogenetic tree was downloaded from MicrobesOnLine (http://microbesonline.org/) (Dehal et al. 2010) and contained 946 species that were annotated by Rfam and used for the reconstruction. We then used the program GLOOME (Cohen and Pupko 2010, 2011) to reconstruct the evolution of each ncRNA family using the MM2 model without branch length optimization. The number of ncRNA families of each group was counted in each direct ancestor of *E. coli*, and their distribution was uploaded to iTOL (http://itol.embl.de) (Letunic and Bork 2007, 2011) for visualization. The evolutionary age of each ncRNA family was determined as the distance on the tree between the first direct ancestor in which the ncRNA family first appeared according to the reconstruction and *E. coli*. The distance is expressed in amino acid substitutions per position, based on the protein sequences used originally to generate the tree. These ages were compared between the *cis*-acting and *trans-*acting ncRNAs using two tailed Mann-Whitney test.

To verify that our conclusions are independent of the Rfam annotation system, we repeated the analysis of the ncRNA families using annotations based on BLAST analysis. We used Standalone BLAST+, version 2.2.28 (Camacho et al. 2009), to search for each ncRNA family in each genome. The genomic sequence of the main chromosome and plasmids of each genome were searched for matches to each *E. coli* ncRNA gene included in the Rfam family. The E-value computation was done while referring to an average genome size of 4 × 10^6^ bases to allow comparison between different genomes. A gene was declared as present in a genome if there was a hit with E-value ≤10 and identity of at least 30% between the tested sequence and the sequence identified by BLAST. We verified that this threshold of 30% does not affect our conclusions: We repeated this annotation applying different identity thresholds (50% and 70%) and obtained consistent conclusions.

### Compiling sBSs, mBSs, and their orthologs

A list of sRNA targets and their binding sites was compiled (Supplemental Table S2 and references therein). This list included sRNAs and targets for which the binding regions between the two were experimentally validated using compensatory mutations, in-vitro probing, or RNase III restriction. Several pairs were excluded for technical reasons; for instance, RybB and McaS were falsely annotated in Rfam, and GlmZ was hard to distinguish from GlmY in the different genomes. This compilation resulted in a total of 60 binding sites included in the analysis. When there were several interactions between a target gene and its regulator sRNA, they were treated as different entries if the binding sites were 10 nt apart on either sRNA or mRNA and were treated as the same interaction if they were closer than that.

The orthologous genes of each target were fetched from the oma-browser database (http://omabrowser.org/, Dec-2012 version) (Altenhoff et al. 2011). This database maps one gene in each genome to a cluster of orthologous genes. The phylogenetic tree taken from MicrobesOnLine (http://microbesonline.org/) (Dehal et al. 2010) was pruned to include genomes that are represented in both the oma database and the Rfam database. The tree was further pruned to exclude the endosymbiont species from the *Enterobacteriales* order. The species *Proteus mirabilis* (TXID: 529507) and two Edwardsiella species (TXID: 498217, 634503) were excluded since their inclusion resulted in inconclusive reconstructions.

The genomic sequences of orthologous genes included in the analysis spanned a region from 150 nt upstream of the translation start site to 50 nt downstream, extracted from the RefSeq database (http://www.ncbi.nlm.nih.gov/refseq/). Orthologous sequences were aligned using the multiple sequence aligner “prank” (Löytynoja and Goldman 2005). The columns of the resulting multiple sequence alignment (MSA) corresponding to the mBS, flanked by 5 nt from each side, were extracted and used to study the evolution of the binding site. The orthologous sRNA sequences were extracted according to the coordinates in the Rfam database and aligned as above. The regions corresponding to the sBS (padded by 5 nt from each side) were extracted for each genome.

Each putative mBS was tested for its functionality according to its hybridization free energy with the orthologous sBS. The free energy was calculated using the RNAduplex program of the Vienna package (Hofacker et al. 1994). If a sRNA could not be identified in a genome, the reconstructed sequence of the sRNA found in the most recent common ancestor of the genomes having the sRNA (obtained by prank) was used to evaluate the binding with the mBS. The free energy of a sRNA–mRNA pairing in *E. coli* was set as a reference. An orthologous target was marked as having a mBS if its interaction free-energy value with the sBS was 0.9 or less of the free energy value in *E. coli*. K12 substrain MG1655. Orthologous targets with higher free-energy or with a gap in the sequence corresponding to the known binding site were marked as lacking the mBS. Genomes lacking an ortholog of the target gene were also marked as lacking the mBS. Binding sites may be missed by the computation because small changes in the sequence might still result in free energy values not passing the threshold. To verify that the free energy threshold used did not affect the results, we repeated the analysis with different thresholds of 0.8 and 0.95, reaching the same conclusions.

### Reconstructing the evolution of a binding site

The binding site evolution was reconstructed on the phylogenetic tree using the GLOOME program (Cohen and Pupko 2010) with the MM2 model without branch length optimization, as above. GLOOME computes the posterior probabilities of gain and loss events along branches of the phylogenetic tree and the posterior probability of ancestral nodes having a trait (here, mBS). The most recent direct ancestor of *E. coli* that had a posterior probability of ≥0.7, where all the ancestors leading from it to *E. coli* had a posterior probability ≥0.7 (to exclude loss event followed by a gain event), was considered the “gain node.” As above, the distance from the gain node to *E. coli* node on the tree was considered as the evolutionary age of the binding site.

## SUPPLEMENTAL MATERIAL

Supplemental material is available for this article.
